# Mitochondrial respiratory chain and Krebs cycle enzyme function in human donor livers subjected to end-ischaemic hypothermic machine perfusion

**DOI:** 10.1371/journal.pone.0257783

**Published:** 2021-10-28

**Authors:** Hamid Abudhaise, Jan-Willem Taanman, Peter DeMuylder, Barry Fuller, Brian R. Davidson

**Affiliations:** 1 UCL Division of Surgery and Interventional Sciences, Royal Free Hospital, London, United Kingdom; 2 Department of Clinical and Movement Neurosciences, UCL Queen Square Institute of Neurology, Royal Free Campus, London, United Kingdom; 3 Organ Recovery Systems, Zaventem, Belgium; Hospital Israelita Albert Einstein, BRAZIL

## Abstract

**Introduction:**

Marginal human donor livers are highly susceptible to ischaemia reperfusion injury and mitochondrial dysfunction. Oxygenation during hypothermic machine perfusion (HMP) was proposed to protect the mitochondria but the mechanism is unclear. Additionally, the distribution and uptake of perfusate oxygen during HMP are unknown. This study aimed to examine the feasibility of mitochondrial function analysis during end-ischaemic HMP, assess potential mitochondrial viability biomarkers, and record oxygenation kinetics.

**Methods:**

This was a randomised pilot study using human livers retrieved for transplant but not utilised. Livers (n = 38) were randomised at stage 1 into static cold storage (n = 6), hepatic artery HMP (n = 7), and non-oxygen supplemented portal vein HMP (n = 7) and at stage 2 into oxygen supplemented and non-oxygen supplemented portal vein HMP (n = 11 and 7, respectively). Mitochondrial parameters were compared between the groups and between low- and high-risk marginal livers based on donor history, organ steatosis and preservation period. The oxygen delivery efficiency was assessed in additional 6 livers using real-time measurements of perfusate and parenchymal oxygen.

**Results:**

The change in mitochondrial respiratory chain (complex I, II, III, IV) and Krebs cycle enzyme activity (aconitase, citrate synthase) before and after 4-hour preservation was not different between groups in both study stages (p > 0.05). Low-risk livers that could have been used clinically (n = 8) had lower complex II-III activities after 4-hour perfusion, compared with high-risk livers (73 nmol/mg/min vs. 113 nmol/mg/min, p = 0.01). Parenchymal pO_2_ was consistently lower than perfusate pO_2_ (p ≤ 0.001), stabilised in 28 minutes compared to 3 minutes in perfusate (p = 0.003), and decreased faster upon oxygen cessation (75 vs. 36 minutes, p = 0.003).

**Conclusions:**

Actively oxygenated and air-equilibrated end-ischaemic HMP did not induce oxidative damage of aconitase, and respiratory chain complexes remained intact. Mitochondria likely respond to variable perfusate oxygen levels by adapting their respiratory function during end-ischaemic HMP. Complex II-III activities should be further investigated as viability biomarkers.

## Introduction

The ongoing donor liver shortage prompted the relaxation of liver acceptance criteria and increased utilisation of high-risk donor or “marginal” livers. Marginal livers are obtained from elderly donors and those with multiple co-morbidities, donation after cardiac death (DCD), steatotic grafts, and livers with prolonged preservation times. These livers are highly susceptible to ischaemia reperfusion injury (IRI) and post-transplant complications [1–13]. New preservation methods were required to improve the outcome and utilisation of these high-risk donor organs.

Mitochondria have a crucial role in organ recovery following transplantation by restoring energy and producing adenine triphosphate (ATP) via oxidative phosphorylation [14]. Conversely, the IRI process is greatly influenced by mitochondria through initiation of oxidative and inflammatory injuries which can lead to cell death and organ failure [8, 15–19]. Oxygen deprivation during ischaemia interrupts mitochondrial oxidative phosphorylation and results in highly reduced electron transport chain (ETC) enzymes [20, 21]. Furthermore, fumarate buildup during ischaemic preservation triggers the reverse activity of mitochondrial complex II and reduction of fumarate to succinate, the latter accumulating progressively and acting as an electron sink [15, 17, 18]. Accumulated succinate is rapidly oxidised upon reperfusion of the stored graft, triggering reverse electron transport (RET) in complex I and causing a burst of mitochondrial reactive oxygen species (ROS) [17, 20, 22, 23]. Prolonged ischaemia could also lead to irreversible and variable damage to ETC enzymes [24–27], but this aspect has not been illustrated in the human liver.

Dynamic organ preservation methods (normothermic and hypothermic machine perfusion) are increasingly advocated for resuscitation and selection of marginal livers. Hypothermic machine perfusion (HMP) has gained a lot of interest recently and clinical observational studies confirmed the safety of end-ischaemic “post-SCS” oxygenated and air-equilibrated HMP. Moreover, end-ischaemic HMP reduced biliary complications and attenuated IRI in marginal livers [28–33]. However, static cold storage (SCS) remains standard practice for liver preservation [34] and there are no clinically validated parameters of liver transplantability in end-ischaemic HMP. Importantly, the exact mechanism of protection in end-ischaemic HMP is not clearly defined.

The protective effect of hypothermic oxygenated machine perfusion (HOPE) was proposed to be secondary to downregulation of mitochondrial respiration and possibly lowered succinate accumulation, leading to mitochondrial stabilisation and an attenuated ROS burst upon reperfusion. These effects were dependent on active perfusate oxygenation [35–41], which was shown to be safe despite earlier studies that warned against the risk of oxidative injury [42, 43]. However, the mechanism behind mitochondrial respiratory depression in HOPE is unknown, and oxygenated HMP in other experimental models increased the oxygen consumption and activated mitochondrial respiration [41, 44–47]. These studies differed in methodology and the measured outcomes, but the inconsistency in mitochondrial respiration results is unexplained. Additionally, air-equilibrated HMP decreased the inflammatory response with reperfusion but there is no evidence to link this to mitochondrial stabilisation [48–50]. Finally, the superiority of actively oxygenated or air-equilibrated perfusion is unknown and both modalities regenerated the depleted ATP and improved mitochondrial function compared with SCS [31, 37, 39, 40, 45, 47, 51–61]. Mitochondrial function has not been directly compared during oxygenated and air-equilibrated HMP in the human liver, and the interplay between perfusate oxygen distribution and local parenchymal uptake are unidentified in the human liver.

This study aimed to examine the feasibility of analysing the activity of mitochondrial ETC and Krebs cycle enzymes during end-ischaemic HMP, comparing arterial, oxygenated and non-oxygenated venous perfusion in a human liver model, and to identify markers of mitochondrial activity which could be evaluated as viability biomarkers. In addition, the delivery efficiency of oxygen to the human liver during end-ischaemic HMP was assessed.

## Materials and methods

### Participants

Ethical approval for the study was obtained from the North London Regional Ethics Committee 3 (reference no. 10/H0709/70) and confirmed with the National Health Service Blood and Transplant (NHSBT) Organ Donor Transplant committee. None of the organ donors was from a vulnerable population and a written informed consent was freely given by the next of kin.

The study involved 44 whole livers, including 38 livers used for mitochondrial assessment and 6 livers used for evaluation of oxygenation kinetics. Livers were procured for human transplantation by different organ procurement teams across the UK between February 2012 and June 2014. All livers had been retrieved using the standard clinical procurement protocols adopted by the UK National Organ Retrieval Service (NORS) [62]. The organs had been deemed unsuitable by the transplant team for clinical use and were subsequently offered for research. Livers were transported from the retrieval hospital to our research centre packed in ice and stored in University of Wisconsin (UW) solution as with routine clinical practice.

Donor variables including age, gender, heart-beating status, cause of death, weight, body mass index (BMI), alanine transaminase (ALT), bilirubin, UK donor liver index (DLI) [63], length of stay in the intensive care unit, warm ischaemic times (WIT), and graft steatosis grading were obtained from the Human Tissue Act-A (HTA-A) forms that accompany procured organs, local transplant coordinators records (manual search and verbally at the time of organ offer), and from the NHSBT Statistics and Clinical Studies Department (personal communication).

The 3 data sources were examined to establish the reasons for not utilising the livers for transplantation. In addition to the reported reasons for non-utilisation, liver characteristics were assessed retrospectively to identify those which would be considered high or low risk grafts as regards their susceptibility to IRI. The distinction between high- and low-risk livers was exclusively based on the reported risk factors and available data, and it cannot be established whether the low-risk livers would have functioned after transplantation. Finally, steatosis was assessed microscopically in haematoxylin and eosin (H&E)-stained wedge biopsies due to the known discrepancy between gross and microscopic steatosis evaluation, as reported by our team [64] and others [65–68].

### Mitochondrial assessment

#### Preservation groups

**Fig 1:**
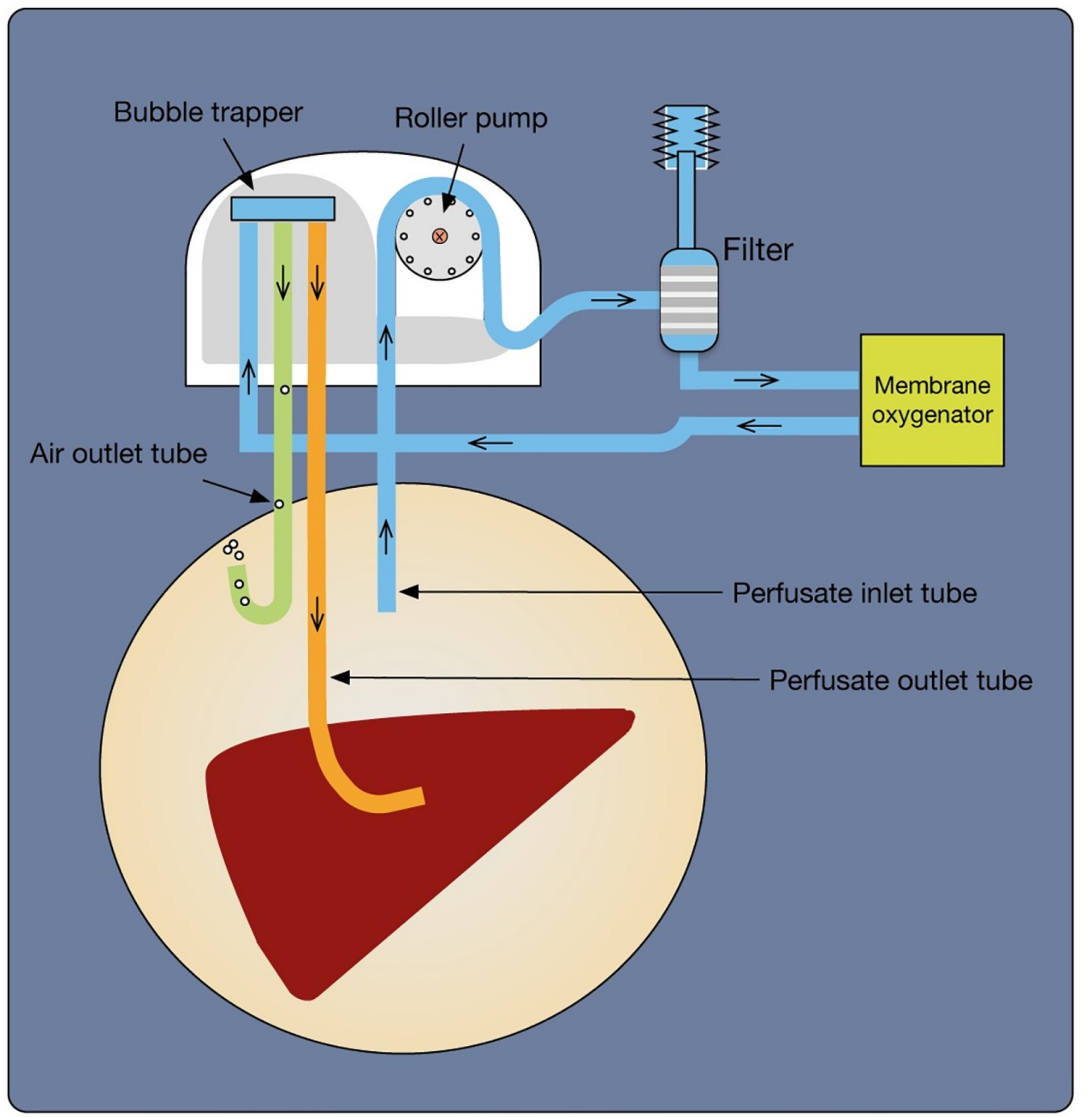
Diagram of the organ recovery workstation^®^, showing the installed membrane oxygenator and perfusate flow.

The Organ Recovery Workstation^®^ prototype, a modified version of the Lifeport Kidney Transporter machine with a dual pumping system (Organ Recovery Systems, Zaventem, Belgium), was used for end-ischaemic single-vessel HMP following the primary SCS period during liver transport. This prototype did not contain an oxygen delivery system, which was integrated for the second stage of the study, Fig 1. Consequently, experiments were performed in 2 stages with randomisation of donor organs between preservation methods at both stages, where each donor organ was utilised for only one preservation group as described previously [69]. Simple donor organ randomisation was performed using a computer-generated list of random numbers and the allocation sequence was concealed in numbered, opaque, and sealed envelopes. Formal sample size calculation was not performed but was guided by feasibility objectives and available resources in line with liver recruitment and anticipated numbers of research-approved livers during the study period.

A total of 38 livers were used for mitochondrial evaluation. Initially, 20 livers were randomised in stage 1 into static cold storage (SCS, n = 6) as a study control, arterial perfusion through the hepatic artery (AP, n = 7), and non-oxygen supplemented venous perfusion through the portal vein (nOVP, n = 7). The SCS group represented continued SCS after the primary SCS period during liver transport, and these livers were submerged in 2 L of Kidney Preservation Solution-1 (KPS-1, Organ Recovery Systems, Zaventem, Belgium) and placed in the icebox for the equivalent period of 4 hours. Although KPS-1 is not usually used for SCS preservation, it was utilised herein to reduce the possible bias from using different preservation solutions. Arterial-only perfusion was assessed following an earlier study by our team, reporting similar endothelial preservation in single- and dual-vessel perfusion [70]. However, we acknowledge that single arterial perfusion is not an established route for clinical liver preservation using HMP.

In stage 2, 18 livers were randomised into nOVP (n = 7) and oxygen supplemented portal vein perfusion (OVP, n = 11). The arterial perfusion group was omitted in this stage as the main focus was on oxygenated vs. air-equilibrated venous perfusion. Donor characteristics and perfusion settings for the nOVP groups in stages 1 and 2 of the study were similar.

#### End-ischaemic HMP

End-ischaemic single-vessel HMP was performed for 4 hours with 2 L of recirculating KPS-1 through the hepatic artery (HA) or portal vein (PV) at 30 mmHg or 7 mmHg, respectively. Perfusion settings were based on previous work by our team [69, 70] and others [71], which were adapted from clinical kidney HMP and resulted in homogenous graft perfusion. For the OVP group, 100% oxygen was administered to the perfusion circuit at a fixed rate of 0.5 L/min using a membrane oxygenator (Dideco Kids D100, Sorin Group Italia, Mirandola, Italy; perfusate pO_2_, 735 mmHg). Hypothermia was maintained at 4–8°C, as per our previous studies [69, 70].

#### Sample collection and mitochondrial isolation

Wedge liver biopsies from segments IV and VII were obtained at the beginning and the end of end-ischaemic HMP or the 4-hour SCS in our laboratory, aiming for > 0.5 g/specimen [25]. As the optimal sampling regime is unknown, we selected these 2 sites and compared them as an indication of heterogeneity. Tissue specimens were immediately snap-frozen in liquid nitrogen and placed on ice until homogenisation (Eurostar digital homogeniser, IKA^®^—Werke GmbH & Co.KG, Staufen, Germany). Mitochondria were isolated by differential centrifugation according to Rickwood et al. [72] and the pellets were suspended in the homogenising buffer and stored at -80°C. Mitochondrial enzyme assays were performed within one week of specimen collection.

#### Spectrophotometric assessment of mitochondrial injury and function

Spectrophotometry assays were performed on mitochondrial fractions of frozen samples rather than fresh tissue due to logistic reasons, and this also precluded the use of polarography, which requires fresh samples [73, 74]. Protein concentrations were used to standardise the mitochondrial enzyme activities to the amount of tissue in the biopsy and were measured using the Pierce BCA Protein Assay Kit (ThermoFisher Scientific, Massachusetts, USA). Citrate synthase was used to evaluate mitochondrial membrane integrity and mass [24, 75–78] and aconitase was used as a marker of irreversible ROS-mediated mitochondrial injury [79–81]. These 2 assays were performed according to Protasoni et al. [82] and Gardner et al. [79], respectively, and each specimen was assayed in quadruplicate. Mitochondrial function was studied by measuring electron transport chain (ETC) enzyme activities. Nicotinamide adenine dinucleotide (NADH):ubiquinone oxidoreductase (complex I), succinate cytochrome c reductase (complexes II & III), and cytochrome c oxidase (complex IV) assays were performed according to the methods of Ragan et al. [83], King et al. [84], and Protasoni et al. [82], respectively, and each specimen was assayed in triplicate.

### Real-time assessment of parenchymal and perfusate oxygen

Separate to the mitochondrial assays above, an additional cohort of 6 sequential livers (non-randomised) were used to evaluate the delivery efficiency of oxygen during HMP. Fibre-optic oxygen microsensors (PreSens GmbH, Regensburg, Germany) continuously recorded oxygen concentrations in the perfusate and parenchyma using a flow-through sensor connected to the PV cannula and a needle-type sensor mounted in a 27-gauge diameter needle, respectively. Before each experiment, the microsensors were calibrated according to the manufacturer’s instructions and the pO_2_ measurements were corrected for temperature and atmospheric pressure.

Standard perfusions were run for 3 hours using PV-only perfusion at 7 mmHg constant pressure. Parenchymal pO_2_ was assessed at 2 representative sites (segments IV and VII) and the intra-liver variation was calculated. To obtain a stable baseline oxygen measurement in perfusate and parenchyma, end-ischaemic HMP was initially run for 30 minutes using air-equilibrated (room air) perfusate. Perfusate was then oxygenated with 100% oxygen at 0.5 L/min for 1.5 hours. After 2 hours of HMP (air-equilibrated and oxygenated perfusion), the oxygen was switched off whilst maintaining pump perfusion for another 1 hour to obtain the dynamics of oxygen depletion. The whole process was divided into 3 phases: baseline pre-oxygenation, active-oxygenation, and post-oxygenation perfusion.

### Statistical analysis

Data were analysed using IBM SPSS Statistics for Macintosh (Version 20.0. Armonk, NY: IBM Corp) and presented as medians and interquartile ranges (IQR) for continuous variables or numbers and percentages for nominal variables. Non-parametric tests were used due to the small sample size and abnormal distribution. For continuous data, Kruskal-Wallis or Mann-Whitney tests were used to compare between groups, and Friedman or Wilcoxon tests were used for within-group comparisons. Categorical data were compared using Chi squared test. The level of significance was set at p < 0.05.

## Results

### Donor graft characteristics

Characteristics of the donor grafts are presented in Table 1. Reported causes of donor death included intracranial haemorrhage (n = 29, 66%), hypoxia (n = 10, 23%) and other causes including respiratory failure, infection, and trauma (n = 5, 11%). The decision to decline grafts for transplantation was multifactorial; the most common reported factor was moderate to severe steatosis (n = 22, 50%), followed by anatomical reasons (n = 7, 16%), fibrosis (n = 6, 14%), poor flow during on-table flush (n = 6, 14%), long WIT (n = 5, 11%), and other factors such as tumours, poor liver function tests, long intensive care stay, and long cold ischaemic time (n = 4 each, 9%). Importantly, 3 livers (7%) were declined due to the lack of a suitable recipient. Over a half (59%) of research livers in this study were obtained from DCD donors, compared to 17% of transplants from DCD donors over the same time period, indicating that non-proceeding donations were disproportionately higher for DCD compared with DBD donors [85]. There were no significant differences in donor graft variables between the preservation groups, Table 2.

**Table 1:** Characteristics of donor liver grafts (n = 44).

Donor characteristics	
Donor age (years)	58 (52–66)
Donor gender	15 females (34%); 29 males (66%)
Donor type	18 DBD (41%); 26 DCD (59%)
Donor weight (kg)	89 (70–95)
Donor BMI	29 (26–33)
ALT (IU/L)	36 (19–72)
Bilirubin (umol/L)	11 (6–16)
UK DLI	1.85 (1.31–2.15)
ICU stay (days)	3 (2–5)
F-WIT (min) ^a^	16 (13–19)
CIT (hr)^b^	12 (10–14)

^a^ For the 26 DCD livers, functional warm ischaemic time (F-WIT) was calculated from the time of sustained drop in systolic blood pressure below 50 mmHg until cold in-situ flush [62].

^b^ Cold ischaemic time (CIT) was calculated from the time of cold in-situ flush until machine perfusion or the 4-hr static cold storage period in our laboratory. ALT, alanine transaminase; BMI, body mass index; DBD, donation after brain death; DCD, donation after cardiac death; ICU, intensive care unit; UK donor liver index (UK DLI) [63].

**Table 2:** Comparison of donor liver characteristics between preservation groups.

	Stage 1				Stage 2		
	SCS	AP	nOVP	Inter-group difference	nOVP	OVP	Inter-group difference
	(n = 6)	(n = 7)	(n = 7)	(p value)	(n = 7)	(n = 11)	(p value)
Age (year)	57 (50–70)	56 (51–65)	57 (49–61)	0.28	62 (48–65)	53 (44–61)	0.25
Gender (male, %)	5, 71%	7, 70%	7, 87%	0.77	6, 66%	8, 72%	0.63
Donor type (DCD, %)	4, 57%	7, 70%	6, 75%	0.85	4, 44%	5, 45%	0.96
Donor weight (kg)	85 (70–90)	87 (79–93)	94 (83–114)	0.36	90 (72–100)	85 (70–94)	0.79
Donor BMI	27 (26–31)	29 (26–35)	31 (25–37)	0.68	30 (25–32)	28 (26–31)	0.79
ALT (IU/L)	20 (17–124)	26 (19–46)	52 (19–73)	0.37	135 (36–304)	36 (22–60)	0.21
Bilirubin (umol/L)	6 (5–17)	14 (6–21)	11 (6–15)	0.28	9 (6–13)	13 (6–17)	0.36
UK DLI	1.47 (1.13–2.10)	1.99 (1.42–2.33)	1.85 (1.31–2.08)	0.76	1.61 (1.04–2.12)	1.54 (1.18–2.13)	0.79
ICU stay (days)	3 (3–5)	2 (2–5)	3 (5–5)	0.43	3 (3–5)	2 (1–5)	0.08
CIT (hr)	11.5 (11.0–12.3)	10.0 (9.8–14.3)	11.5 (7.0–13.8)	0.65	10.0 (10.0–14.0)	13.0 (12.0–15.0)	0.16
Steatosis % ^a^	2 (0–15)	6 (5–17)	2 (1–7)	0.31	4 (0–19)	6 (4–21)	0.36
F-WIT (min)	18 (14–26)	16 (15–30)	17 (13–22)	0.82	16 (12–26)	17 (12–28)	0.86

^a^ Steatosis is expressed as the percentage of macrosteatosis in liver biopsies that were retrospectively examined by 2 pathologists at our institute. ALT, alanine transaminase; BMI, body mass index; CIT, cold ischaemic time; DCD, donation after cardiac death; F-WIT, functional warm ischaemic time of livers from donors after cardiac death in each group; UK-DLI, UK donor liver index.

### Mitochondrial injury and function

#### Stage 1

**Fig 2:**
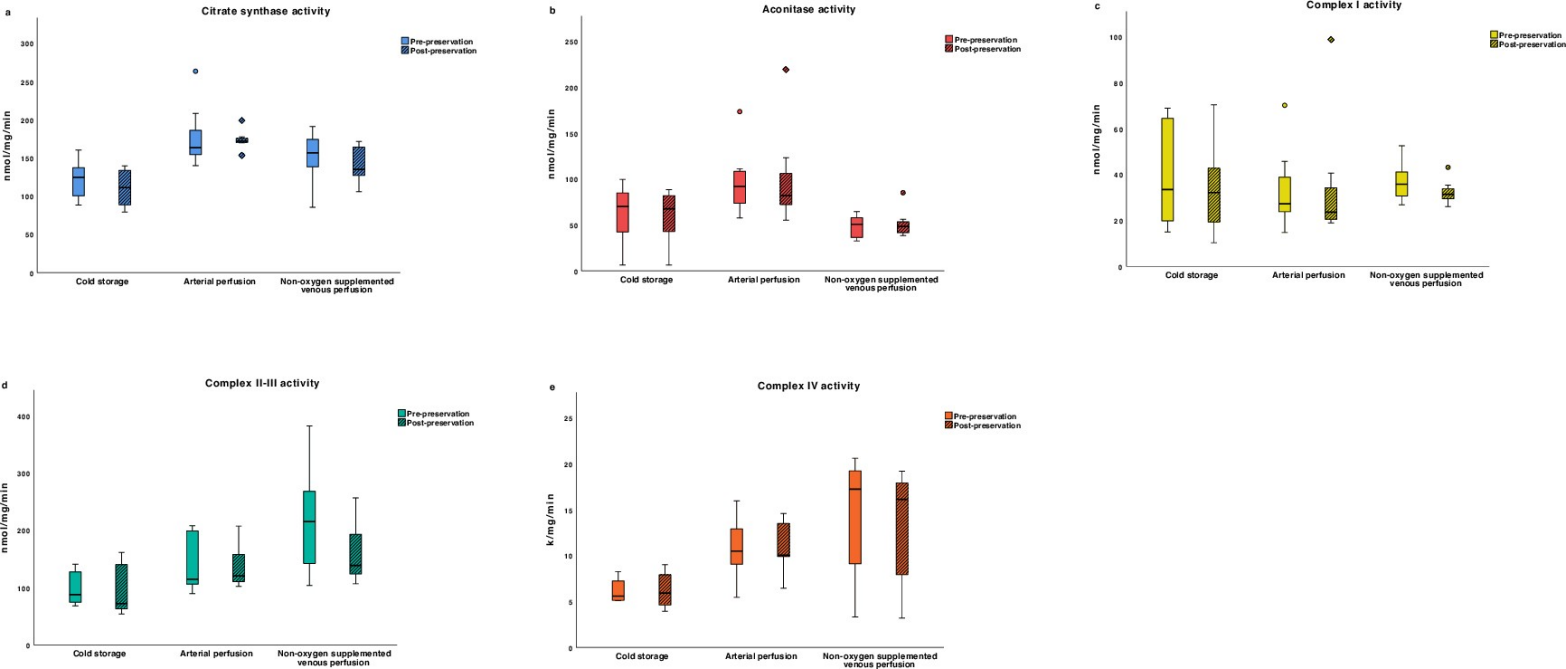
Box whisker plots of mitochondrial enzyme activities in stage 1. The plots demonstrate the distribution of enzyme activities round the median in each group, shown before and after 4-hr end-ischaemic hypothermic machine perfusion and 4-hr static cold storage. The change in enzyme activities was not significantly different between groups (p > 0.05). Top and bottom whiskers represent the maximum and minimum values, respectively. Circles represent outliers (1.5 to 3x interquartile range) and diamonds represent extreme outliers (> 3x interquartile range).

The activities of citrate synthase, aconitase, and ETC enzymes I to IV were compared between SCS (n = 6), AP (n = 7), and nOVP (n = 7) livers. The change in mitochondrial Krebs cycle and ETC enzyme functions were not significantly different after 4-hour end-ischaemic HMP compared with 4-hour SCS preservation (p > 0.05), Fig 2.

#### Stage 2

**Fig 3:**
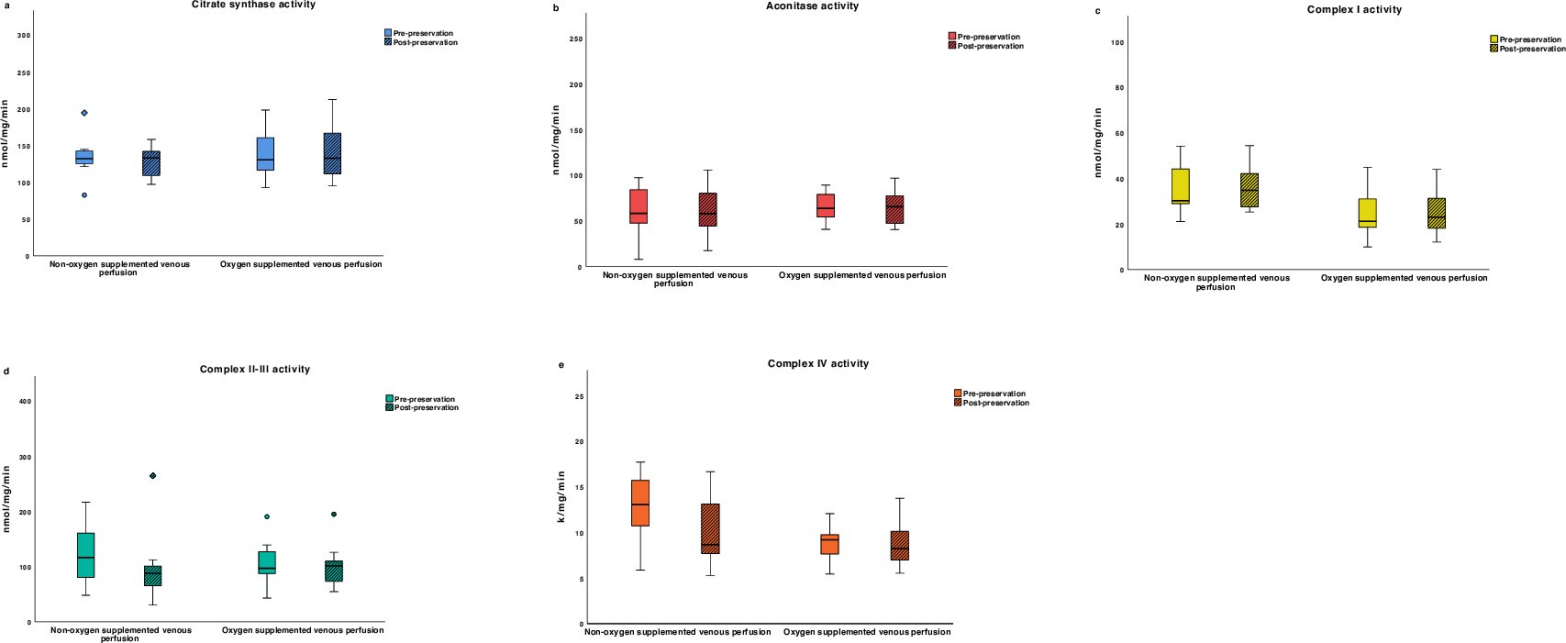
Box whisker plots of mitochondrial enzyme activities in stage 2. The plots demonstrate the distribution of enzyme activities round the median in each group, shown before and after 4-hr end-ischaemic hypothermic machine perfusion. The change in enzyme activities was not significantly different between preservation groups (p > 0.05). Top and bottom whiskers represent the maximum and minimum values, respectively. Circles represent outliers (1.5 to 3x interquartile range) and diamonds represent extreme outliers (> 3x interquartile range).

The mitochondrial enzyme activities were compared between nOVP (n = 7), and OVP (n = 11) livers. The change in mitochondrial Krebs cycle and ETC enzyme functions were not significantly different between the 2 groups (p > 0.05), Fig 3.

#### Mitochondrial assessment in low-and high-risk livers

Out of 38 livers included in this part of the study, 8 livers were considered to have a low risk of IRI, Table 3. These livers were randomly allocated to the PV perfusion groups. In stage 1, 2 livers were allocated to the nOVP group. In stage 2, 1 liver was allocated to the nOVP group and 5 livers were allocated to the OVP group.

**Table 3:** Reasons of graft decline for transplantation and characteristics of low-risk livers.

Case	Reason 1	Reason 2	Reason 3	Other reasons ^a^	Microscopic steatosis assessment ^b^	Donor age	Donor gender	Donor type	Donor weight (kg); BMI	ALT (IU/L)	Bilirubin (umol/L)	UK DLI	ICU stay (days)	F-WIT (min)
1	Other	Moderate steatosis	Research ^c^	No suitable recipient	None	61	Male	DBD	90; 29	74	16	1.12	4	NA
2	Anatomical	Research ^c^	NR	No suitable recipient, subcapsular haematoma	Mild	45	Male	DCD	99; 37	135	10	2.04	6	12
3	Mild steatosis	Research ^c^	NR	Mild steatosis, capsular laceration	Mild	61	Male	DCD	90; 31	31	9	2.14	4	17
4	Tumour	Research ^c^	NR	Moderate steatosis	None	58	Female	DBD	90; 33	60	13	1.19	2	NA
5	Other	Research ^c^	NR		Mild	44	Male	DBD	94; 28	22	14	0.87	1	NA
6	Tumour	NR	NR	Liver & kidneys lesions	None	57	Female	DCD	55; 20	18	6	2.06	5	15
7	Infection	Moderate steatosis	Research ^c^	Small bowel perforation	None	52	Female	DCD	70; 27	72	4	2.09	6	11
8	Moderate steatosis	Research ^c^	NR	No suitable recipient	Mild	81	Male	DBD	95; 28	36	16	1.24	1	NA

These livers were identified retrospectively based on data collected from the NHSBT register, coordinators records, and HTA-A forms. Reasons 1, 2, 3 are the primary, secondary, and tertiary reasons for rejecting the liver as per the NHSBT register.

^a^ Other reasons as obtained from HTA-A forms and liver coordinator records.

^b^ Microscopic steatosis assessment was performed retrospectively on H&E-stained parenchymal sections by 2 pathologists at our institute. Steatosis was graded as normal (none), mild, moderate, and severe.

^c^ Research, as a reason for graft decline, was recorded to keep track of the livers offered for research and was not a reason for declining grafts in itself; livers were only offered for research after being rejected for clinical use by all UK transplant centres. Cold ischaemic time was < 12 hours for low-risk livers (n = 8). ALT, alanine transaminase; DBD, donation after brain death; DCD, donation after cardiac death; F-WIT, functional warm ischaemic time; NA, not applicable; NR, not reported.

Post-preservation complex II-III activities were significantly lower in low-risk livers (73 nmol/mg/min (56–101) vs. 113 nmol/mg/min (89–160), p = 0.01). There was no significant difference in the remaining enzymes at any time point, including the initial pre-preservation (baseline) levels and the change in enzyme activities between low- and high-risk livers (p > 0.05), Table 4.

**Table 4:** Mitochondrial enzyme activity in low- and high-risk livers.

Enzyme activity	Low-risk (n = 8)		High-risk (n = 30)		Intergroup difference (p-value) ^a^
	Pre-preservation	Post-preservation	Pre-preservation	Post-preservation	
**Citrate synthase**	134 (119–179)	134 (110–149)	141 (120–163)	136 (113–171)	0.74, 0.45, 0.31
**(nmol/mg/min)**					
**Aconitase**	55 (47–66)	53 (43–73)	66 (47–90)	70 (45–86)	0.23, 0.33, 0.45
**(nmol/mg/min)**					
**Complex I activity**	28 (21–40)	25 (21–33)	30 (21–45)	29 (21–41)	0.63, 0.37, 0.25
**(nmol/mg/min)**					
**Complex II-III activity**	106 (56–136)	73 (56–101)	118 (93–196)	113 (89–160)	0.19, 0.01*, 0.17
**(nmol/mg/min)**					
**Complex IV activity**	9 (7–13)	8 (7–10)	9 (6–14)	9 (6–14)	0.90, 0.79, 0.54
**(k/mg/min)**					

Mitochondrial enzyme activities were measured before and after preservation (4-hr end-ischaemic HMP and 4-hr SCS) and compared between low- and high-risk livers.

^a^ Intergroup difference, presented as p-value, was calculated for pre-preservation, post-preservation, and change in enzyme activities.

* denotes a significant intergroup difference.

#### Mitochondrial intra-liver heterogeneity

**Fig 4:**
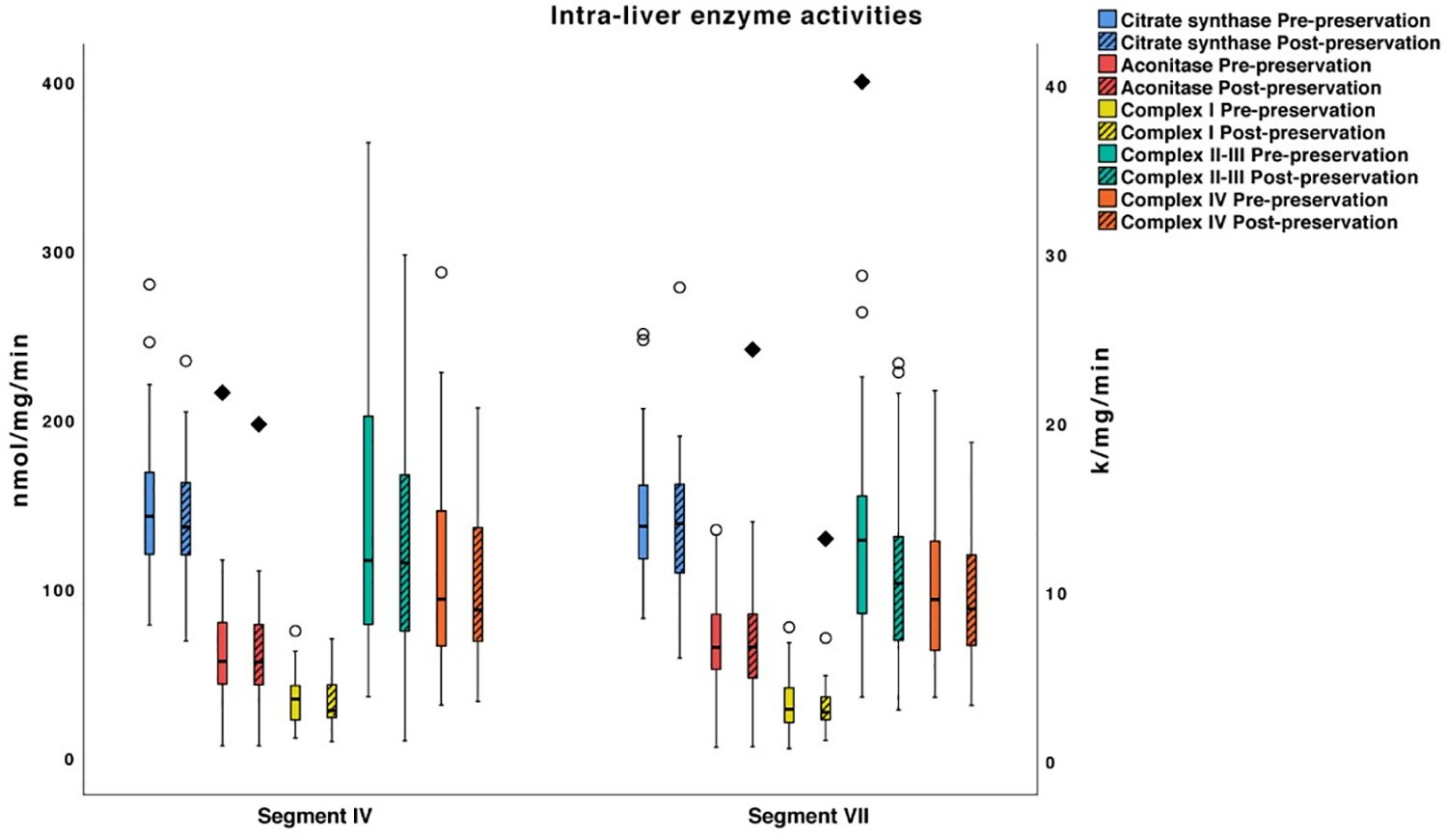
Box whisker plots of mitochondrial enzyme activities in liver segments IV and VII. The plots demonstrate the distribution of enzyme activities round the median in each segment, shown before and after 4-hr preservation. The enzyme activities were not significantly different between segments IV and VII (p > 0.05). The activities of citrate synthase, aconitase, complex I-III are expressed in nmol/mg/min while the activity of complex IV is expressed in k/mg/min. Top and bottom whiskers represent the maximum and minimum values, respectively. Circles represent outliers (1.5 to 3x interquartile range) and diamonds represent extreme outliers (> 3x interquartile range).

The change in mitochondrial Krebs cycle and ETC enzyme functions before and after preservation were compared between segments IV and VII. There was no significant difference in any of the parameters studied (p > 0.05), Fig 4.

### Perfusate and parenchymal oxygen concentrations

**Fig 5:**
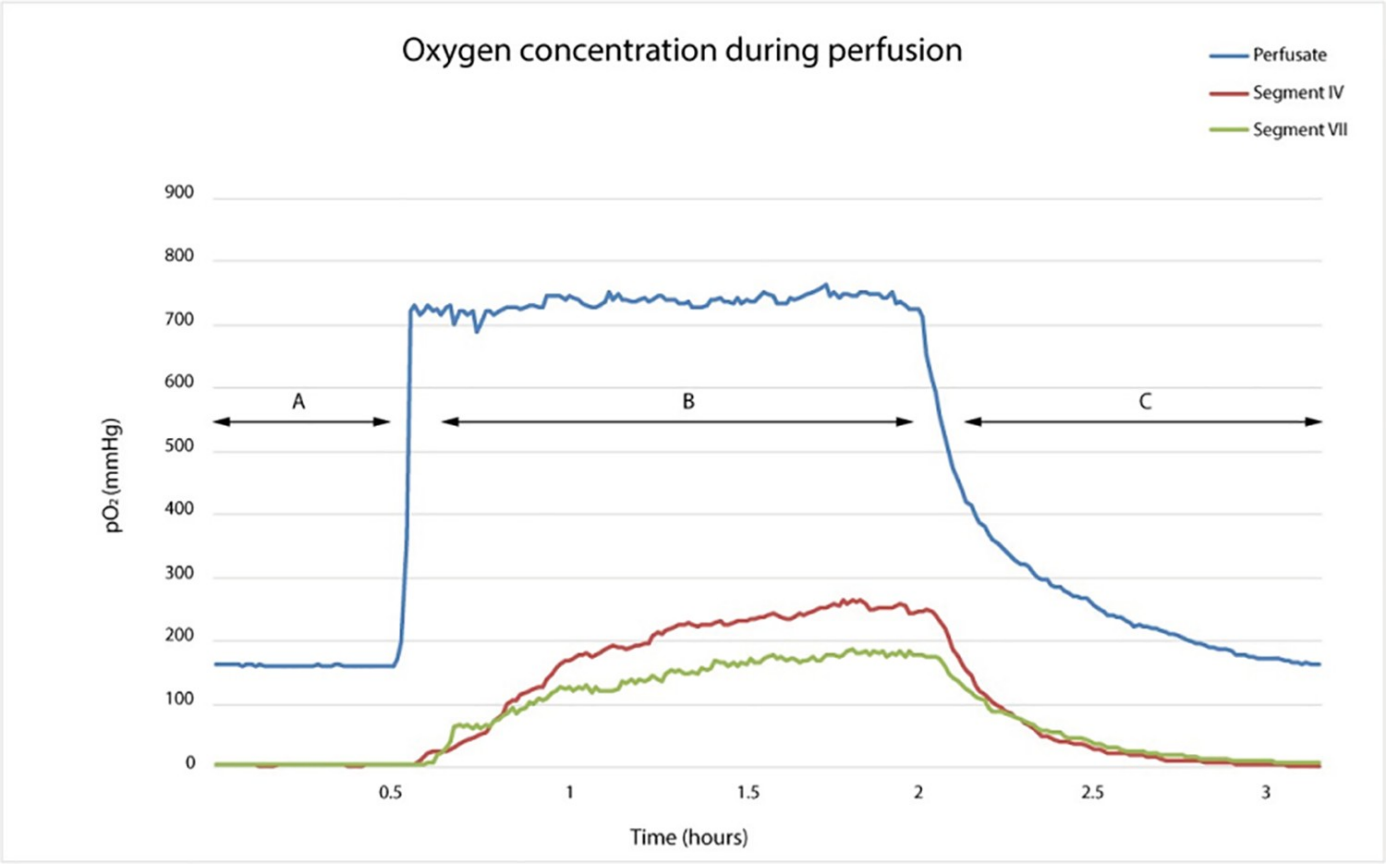
Oxygen concentration during end-ischaemic HMP. This figure shows the pO_2_ in the perfusate and segments IV and VII during (A) pre-oxygenation, (B) active oxygenation, and (C) post-oxygenation phases.

The parenchymal pO_2_ was consistently lower than perfusate pO_2_ in all 3 perfusion phases (baseline pre-oxygenation, active-oxygenation, and post-oxygenation perfusion, p ≤ 0.001), Fig 5. Similar to parenchymal oxygen levels, perfusate oxygen concentrations increased significantly during oxygenated perfusion compared to pre-oxygenation and post-oxygenation phases (p < 0.001). Rising pO_2_ levels in the parenchyma were detected within 5 minutes of starting active perfusate oxygenation. A steady state parenchymal pO_2_ was achieved on average in 28 minutes compared to the perfusate, which stabilised within a few minutes (3 vs. 28 minutes, p = 0.003). The time required for pO_2_ to return to baseline levels after cessation of active oxygenation was longer in the perfusate compared to the parenchyma (75 vs. 36 minutes, p = 0.003).

When intra-liver variation was assessed, pO_2_ was lower in segment VII compared to segment IV during all 3 phases: pre-oxygenation, 0.6 mmHg (0.1–2.5) vs. 4.5 mmHg (0.2–6.4); active oxygenation, 179.9 mmHg (107.6–189.8) vs. 243.1 mmHg (149.9–312.8); post-oxygenation, 0.9 mmHg (0.2–2.8) vs. 4.8 mmHg (0.1–6.9). However, the difference was not statistically significant between the 2 liver segments (p > 0.05 for all 3 phases).

## Discussion

This is the first study to analyse the activity of individual ETC and Krebs cycle enzymes and to report on parenchymal oxygen concentration during end-ischaemic HMP in the human liver.

Oxidative damage to mitochondria is one of the main concerns with active oxygenation during HMP. Aconitase is a sensitive mitochondrial biomarker of irreversible oxidative injury by superoxide or hydrogen peroxide [79–81]. Based on our results, there was no evidence that highly-oxygenated perfusate caused more oxidative injury than air-equilibrated perfusion or SCS, as evidenced by similar aconitase measurements in all groups. Our results in human end-ischaemic HMP are consistent with animal studies showing minimal oxidative damage during SCS or SCS followed by HMP, and more specifically, the lack of oxidative damage when high-oxygen flow is introduced into the perfusate [39, 86]. Additionally, mitochondrial mass and membrane integrity were preserved in all groups, as shown by stable mitochondrial citrate synthase activity [24, 75–78].

There was no significant change in isolated ETC enzyme activities over the time course of end-ischaemic perfusion as compared to SCS preservation, indicating stability of this part of mitochondrial respiration and confirming the lack of oxidative damage to respiratory chain enzymes. Mechanistic assessment of mitochondrial function during HOPE reported reduced mitochondrial respiration based on decreasing perfusate NADH and pCO_2_ in a porcine model [35]. However, it was not demonstrated whether the decreased NADH concentration was due to increased oxidation (consumption) or decreased production. In addition, pCO_2_ was reported to increase initially and was consistently higher than deoxygenated perfusion [35], indicating that mitochondrial respiration was activated, at least initially. Other experimental models show contradicting results of increased oxygen consumption and active mitochondrial respiration following oxygenated HMP [44–46].

The reason for the discrepancy in mitochondrial activity between previous experimental models and the current study is unclear but could be related to differences in endpoints, methodology, and species [87, 88]. Importantly, the current study is the only one to report on mechanistic changes in ETC enzymes during end-ischaemic HMP of human discard livers. The lack of interaction between mitochondria and cytosol in isolated mitochondrial assays could also be relevant in this study; mitochondrial complex I and possibly complex II undergo a conformational change between an active and inactive form depending on the cellular state and substrate availability, without affecting the abundance of the enzymes [89]. Recent evidence also indicates that complex I inactivation after reperfusion is associated with reversible dissociation of the flavin mononucleotide cofactor from complex I [90], suggesting that the enzyme activity can be maintained if suitable circumstances are provided. More mechanistic studies on mitochondrial alterations during end-ischaemic HMP are required to enhance our understanding of the complex mitochondrial machinery in preserved organs.

The discrepancy in the mitochondrial results outlined above might indicate that other mechanisms are responsible for the proposed protective effect of end-ischaemic HMP. A possible mechanism of mitochondrial protection during end-ischaemic HMP could be related to a reversible and transient increase in mitochondrial respiration at the start of end-ischaemic HMP, leading to consumption (oxidation) of the reducing substances and stabilisation of the ETC enzymes with subsequent dampening of the inflammatory response. This hypothesis agrees with previous evidence indicating that mitochondrial inactivity and abundance of electrons from reducing substrates are the main triggers of the ROS burst via reverse electron transport, which is abolished in the presence of active mitochondria [20]. Mitochondrial assessment in the current study was restricted to 2 time points only which may not have adequately reflected real-time changes in mitochondrial activity. Future research should aim to measure mitochondrial function at more frequent intervals or in real-time.

There is a consensus that increased reverse activity of complex II during ischaemia with subsequent accumulation of succinate are necessary for the ROS burst associated with reperfusion [17, 20, 22]. Our results show greater post-perfusion complex II-III activity in high-risk marginal livers, which suggests that this group has a higher potential to generate ROS upon reperfusion. This finding has not been reported previously in the context of liver preservation, including end-ischaemic HMP. As such, a more detailed analysis of complex II activity and succinate levels could provide new biomarkers for graft viability assessment.

Another research area which should be explored for possible therapeutic interventions is methods to decrease succinate accumulation during ischaemia or succinate oxidation upon reperfusion. Recent studies in animal models of stroke and myocardial infarction have shown promising results for mitochondria-targeted therapy [17, 91–94]. HMP could allow mitochondrial manipulation during the critical time of ischaemic succinate accumulation, which provides a promising opportunity for protection against IRI. This could provide an advantage over normothermic machine perfusion which has not shown evidence of mitochondrial protection and is also associated with a variable degree of reperfusion injury [95].

The optimal number of specimens or biopsies required for accurate mitochondrial activity assessment during human liver preservation is unknown. Intra-liver heterogeneity was examined here in central (segment IV) and peripheral (segment VII) parenchymal sites, and there were no significant differences. This implies that the 2 specimens were representative of the whole liver and that one biopsy is adequate for spectrophotometric analysis of mitochondrial enzymes in future studies.

Parenchymal oxygen was lower in right lobe segment VII compared to central segment IV, but the difference was not significant, possibly due to type II error. This segmental variation in oxygen tensions has not been described previously during end-ischaemic HMP, and could be related to gradual oxygen consumption with perfusate moving distally or due to variation in hepatic blood flow between liver segments [96, 97], which was not directly assessed here. It is unlikely that the gradual decrease in oxygen concentration was associated with inadequate oxygen supply distally, based on similar mitochondrial function and injury between the 2 liver segments.

Parenchymal pO_2_ was consistently lower than perfusate pO_2_ during oxygenated and air-equilibrated perfusion, and parenchymal pO_2_ dropped significantly faster after cessation of oxygen flow. It is unlikely that the lower parenchymal oxygen was due to poor organ perfusion, as these livers perfused homogenously on gross examination and perfusion dynamics improved throughout perfusion as described previously by our team [69]. Air-equilibrated perfusion in the absence of an oxygen carrier in the perfusate was associated with consumption of almost all available oxygen, with a median parenchymal pO_2_ < 5 mmHg in central and peripheral liver segments. This is a reflection of the large liver size and dense parenchyma in the human liver and active basal metabolism. Collectively, these results imply active metabolism and the necessity of oxygen during end-ischaemic HMP of the liver, in line with previous studies [35, 71, 98–101].

The parenchymal oxygen levels increased within 5 minutes of commencing active oxygenation, which agrees with experimental studies in the HOPE model that reported graft perfusion within 5 minutes of commencing perfusion with a fluorescein-stained perfusate [102]. Altogether, these findings indicate that end-ischaemic HMP is capable of delivering oxygenated perfusate to the tissues within a short perfusion time. Despite early oxygen diffusion to the parenchyma, there was a time lag on average of 28 minutes (range up to 44 minutes) between commencing active perfusate oxygenation and achieving stable parenchymal oxygen levels. This lag period suggests that oxygenated perfusion triggered initial mitochondrial respiration with increased oxygen consumption, as identified by the upward slope of parenchymal oxygen, which was followed by stabilisation of mitochondrial respiration producing the parenchymal oxygen plateau. These observations indicate the ability of mitochondria to respond to oxygen alterations in the medium by changing their respiratory function during hypothermia. Notably, the change in parenchymal oxygen was evident following the initiation of oxygen, but this does not rule out a lower baseline mitochondrial activity in the air-equilibrated medium, as shown by the lower parenchymal pO_2_ compared to the perfusate.

This study presented a few limitations. First, the liver grafts studied had been declined for clinical transplantation and hence there is no information on how mitochondrial evaluation would have equated with post-transplant graft function. However, results from this research and other preclinical studies could provide essential information for refining diagnostic tools and can be implemented for designing future clinical protocols. Second, the study was not powered to assess efficacy of end-ischaemic HMP and a formal sample size calculation was not done. Steps were taken to improve the power and precision within the limits of the anticipated liver recruitment and study design, including the analysis of paired measurements of continuous variables and measuring the enzyme activity in triplicate or quadruplicate for each specimen. Third, the 4-hour preservation period after the primary SCS might have been insufficient to detect differences in ETC enzyme activities; functions of ETC complexes II, III, IV and mitochondrial respiration were maintained up to 24 hours in cold-stored rodent and pig livers [24, 25]. Fourth, more information on mitochondrial respiration could have been obtained by additional polarographic and adenine nucleotide assays in freshly isolated mitochondria or cells, but these tests were not performed due to logistic reasons. Future research should include these analyses in addition to measurement of mitochondrial electron donors and carriers. Newer methods of mitochondrial function assessment in intact cells using fluorescence microscopy could also prove valuable in this context [103]. Finally, an additional period of warm reperfusion could have been beneficial for examining the endpoints following warm reperfusion injury. However, isolated reperfusion would not provide data similar to the clinical scenario due to the absence of circulating leukocytes and platelets.

In summary, spectrophotometric evaluation of mitochondrial injury and function in end-ischaemic HMP of the human liver is feasible. We found preliminary evidence that actively oxygenated and air-equilibrated end-ischaemic HMP did not induce mitochondrial oxidative damage during the ischaemic period, and that mitochondrial respiratory chain complexes remained intact during cold preservation for 4 hours. Mitochondria likely respond to variable oxygen levels in the perfusate by adapting their respiratory function during end-ischaemic HMP. Besides, there was evidence of early oxygen diffusion and possible site variation in parenchymal oxygen levels. Measurement of complex II-III activities might identify biomarkers for graft quality assessment and should be further investigated in perfused organs destined for clinical use in sufficiently-powered trials. Future research should aim to identify the optimal oxygen supply during end-ischaemic HMP of the human liver and to verify if certain marginal livers are better protected by oxygenated or air-equilibrated hypothermic perfusion. Addressing these questions will consequently affect the design of future organ perfusion systems and the outcome of liver transplantation. Finally, more studies are required to investigate novel mitochondria-targeted therapeutics.

## Supporting information

S1 Data (XLSX)
